# Properties of AgSnO_2_ Contact Materials Doped with Different Concentrations of Cr

**DOI:** 10.3390/ma15144793

**Published:** 2022-07-08

**Authors:** Jingqin Wang, Jingting Xu, Yancai Zhu, Delin Hu, Ningyi Lu, Defeng Cui, Peijian Guo

**Affiliations:** 1 State Key Laboratory of Reliability and Intelligence of Electrical Equipment, Hebei University of Technology, Tianjin 300130, China; xjt13072055026@sina.com (J.X.); zhuyancai@hebut.edu.cn (Y.Z.); 2 Suzhou Electrical Apparatus Science Academy Co., Ltd., Suzhou 215104, China; eservice@eeti.com.cn; 3 Xiamen Hongfa Electroacoustic Co., Ltd., Xiamen 361021, China; lu_ny@hongfa.cn; 4 Guilin Electrical Equipment Scientific Research Institute Co., Ltd., Guilin 541004, China; cuidf10@126.com; 5 Tianjin Research Institute of Electric Science Co., Ltd., Tianjin 300300, China; guopeijian@tried.com.cn

**Keywords:** AgSnO_2_ contact material, doping, elastic constant, arc energy

## Abstract

As an important component carrying the core function and service life of switching appliances, the selection and improvement of electrical contact materials is of great significance. AgSnO_2_, which is non-toxic, environmentally friendly and has excellent performance, has become the most promising contact material to replace AgCdO. However, it has deficiencies in machinability and electrical conductivity. The property of AgSnO_2_ contact material was improved by doping element Cr. The relationship between the mechanical and electrical properties of AgSnO_2_ contact materials and doping concentrations were investigated and analyzed by simulation and experiment. Based on the first principle, the elastic constants of supercell models Sn_1−x_Cr_x_O_2_ (x = 0, 0.083, 0.125, 0.167, 0.25) were calculated. The results show that the material with a doping ratio of 25% is least prone to warp and crack, and the material with a doping ratio of 12.5% has the best toughness and ductility and the lowest hardness, which leads to molding and is subsequently easier to process. The Cr-doped AgSnO_2_ contacts with different doping proportions were prepared by the sol–gel and powder metallurgy method. Additionally, their physical performance and electrical contact properties were measured in experiments. The results show that the doped SnO_2_ powders prepared by the sol–gel method realize integration doping, which is consistent with the crystal model constructed in the simulation calculation. Sn_0.875_Cr_0.125_O_2_ has lower hardness, which is beneficial to process and form. Doping helps to stabilize the arc root, inhibit the ablation of contact by arc, reduces arc duration and arc energy, improves the resistance to arc erosion of AgSnO_2_ contact material, and makes electrical contact performance more stable. The contact material with a doping concentration of 16.7% has the best arc erosion resistance.

## 1. Introduction

The electric contact undertakes the functions of breaking and connecting circuits in a switching apparatus. The operation reliability and service life of various electrical appliances depend on the working performance and quality of contacts [1]. Silver has the best conductivity (63 × 10^6^ (m·Ω)^−1^) and thermal conductivity (429 W/m·K) of all metals, but is inferior in its resistance to welding and arc erosion. Silver-based electrical contact material, which adds a reinforcing phase in Ag matric, can improve arc breaking performance, voltage withstand capacity and machining performance on the basis of its excellent conductivity and thermal conductivity, and meet the more stringent and complex use requirements of modern industry [2,3]. Therefore, it is deeply favored in the electrical contact industry. AgMeO has attracted wide attention therein. Metal oxide as a reinforcing phase can significantly improve the resistance to fusion welding and arc burning of silver-based contact materials, among which the most representative are AgCdO and AgSnO_2_. AgCdO releases toxic Cd vapor during use. The European Union issued RoHS and WEEE directives to limit the use of cadmium and other harmful substances in electronic and electrical equipment [4,5]. AgSnO_2_ is non-toxic, environmentally friendly and has excellent performance, and is fast becoming the most promising contact material to replace AgCdO. However, the second phase SnO_2_ is a wide band gap semiconductor material, which is almost insulated. Under the action of long-term arc, the oxide particles that are hard to decompose gradually precipitate and cover on the contact surface, resulting in a decline in conductivity, a rise in temperature, intensification of arc ablation degree, deterioration of surface condition, and the formation of a vicious circle, affecting the service life of electrical appliances. In addition, SnO_2_ particles have high hardness and brittleness. AgSnO_2_ materials prepared by traditional methods have poor plasticity and ductility. Cracks easily occur in the preparation process, which results in difficulties of material forming and subsequent processing and limits the application range of AgSnO_2_ contact materials [6,7,8].

Professor Rieder proposed that the main factors determining the properties of AgMeO electrical contact materials are the manufacturing process and doping additives [9]. Therefore, at present, regarding the problem of the insufficient performance of AgSnO_2_, researchers mainly conduct in-depth research from two aspects: improved preparation process and doping modification [10,11]. This paper mainly studies the effect of doping on the properties of AgSnO_2_. The commonly used doping additives are mainly divided into metal element additives and non-metallic element additives [12].

The research shows that metal elements can effectively improve the wettability between liquid Ag and SnO_2_ particles, enhance the adhesion ability of the solid–liquid interface, suspend oxide particles in molten Ag, improve the viscosity of liquid Ag and reduce the temperature rise in contact surface under arcs to improve the resistance to fusion welding and arc burning of contact materials and prolong their electrical life [13]. In the process of preparing AgSnO_2_ contact material using the internal oxidation method, Doduco company added W, Bi, Sb, Mo, Zn and other elements and analyzed them using a differential thermal analyzer (DTA) and thermo gravimetric analyzer (TGA). The metal oxide generated by the reaction could effectively improve the thermodynamic stability of contacts and the wettability of liquid Ag on the surface of SnO_2_. The microstructure of the melting zone after the action of arcs showed that the oxide particles were tiny and dispersed; agglomeration did not happen in microscopic structure. Additionally, a lean oxide layer did not exist inside [14]. Reference [15] discussed the effects of metal oxides Co_2_O_3_, Sb_2_O_3_, Cr_2_O_3_ and CuO on the physical properties and electrical contact properties of AgSnO_2_ contact materials. The results showed that CuO and Cr_2_O_3_ as additives were beneficial to improve the wettability and compactness of the material, inhibited the material transfer and splash loss between the two poles, and reduced the area of the material eroded by the arcs. Co_2_O_3_ and Sb_2_O_3_ had significant impacts on the mechanical properties, especially the hardness and tensile strength. The tensile capacity of the material was enhanced. Reference [16] calculated the electronic structure and magnetic moment characteristics of SnO_2_ doped with transition metal elements (V, Cr, Mn). The results showed that the bond length between transition metal elements and O elements became shorter and O atoms had a tendency to move to the transition metal atom. After doping V and Cr, obvious spin polarization phenomenon appeared near the Fermi level, showing semi-metallic properties.

Most rare earth elements belong to metal elements containing a unique 4f sublayer electronic structure, with an unfilled outermost and sub outer electronic structure, which can provide a variety of electronic energy levels and active chemical properties [17]. According to studies in recent years, adding rare earth elements to AgSnO_2_ contact materials, the oxide generated under the action of high temperature can be suspended in the molten Ag pool to improve the viscosity, reduce material transfer, and ameliorate the electrical and mechanical properties of contact materials, and therefore is widely valued by researchers [18].

Non-metallic elements often contain p orbitals that are unfilled with electrons, while the valence band of SnO_2_ is mainly contributed by O 2p orbitals. Therefore, doping non-metallic elements will aggravate the hybridization between orbitals, move the valence band of SnO_2_ to a higher energy level, reduce the band gap width, increase the carrier transition probability and improve the conductivity [19].

For now, improving the performance of AgSnO_2_ contact materials by doping is still in the experimental stage. How to obtain the best performance depends on the original experiments and experiences, which waste money and energy. Therefore, a combination of simulation and experiment is adopted in this paper. The metal element Cr, as the doping element, can reduce the internal pores of a material to improve compactness and conductivity relatedly. However, at present, the selected doping elements are mostly rare earth elements. The relevant literature concerning Cr-doped AgSnO_2_ contact materials is relatively rare and pays more attention to its magnetism or only concerns experiments carried out without studying the influence of doping theoretically. More than that, there is no literature concerning the study of the effect of Cr doping concentration on the mechanical properties and electrical contact properties of AgSnO_2_ contact material in the field.

In this paper, firstly, based on the first principle, the mechanical properties of SnO_2_ supercells doped with different concentrations of Cr were simulated and calculated, and the influence of doping concentration on machining performance was analyzed by elastic modulus. Secondly, the AgSnO_2_ contact materials doped with different concentrations of Cr were prepared using the sol–gel method, high-energy ball milling and powder metallurgy method combined. Finally, the physical and electrical contact properties of contact materials were studied by experiments.

## 2. Materials and Methods

The workflow of this study can be divided into two sections, i.e., the simulation calculation of the elastic constant of SnO_2_, and the physical property test and electrical contact test of AgSnO_2_ electrical contact material.

### 2.1. Cell Model and Calculation Methods

Since SnO_2_ is the main component that determines the properties of AgSnO_2_ contact materials, the elastic constants of SnO_2_ lattices were calculated to simulate the mechanical properties of AgSnO_2_.

**Figure 1:**
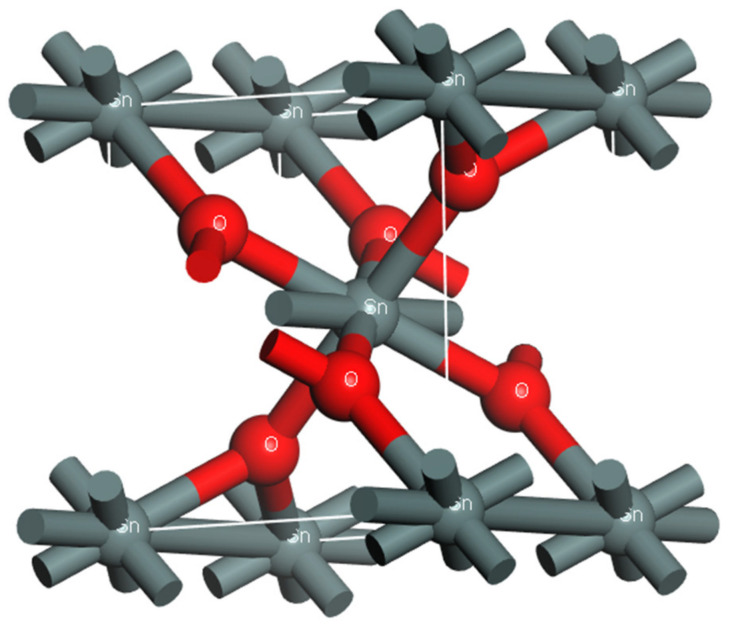
Cell model.

SnO_2_ has a tetragonal rutile structure, which belongs to a body-centered tetragonal system. The lattice constants are *a* = *b* = 4.737 × 10^−8^ m, *c* = 3.816 × 10^−8^ m. As shown in Figure 1, each SnO_2_ unit cell contains two Sn atoms and four O atoms [20]. Because the high doping concentration led to a decrease in carrier mobility, the doping concentration was controlled to below 50%. In order to replace the Sn atom in the supercell model after cell expansion with the Cr atom and realize the atom substitution doping method, the doping concentration selected in this paper was set as 0, 8.3%, 12.5%, 16.7%, 25%. The supercell model Sn_1−x_Cr_x_O_2_ (x = 0, 0.083, 0.125, 0.167, 0.25) was constructed by the atomic substitution method in Materials Studio software. Table 1 shows the relationship between the doping ratio and super cell.

Based on the first principle of density functional theory (DFT), the simulation was carried out using the CASTEP module. Considering the non-uniformity of electron density in the real system, the exchange correlation between electrons was dealt by the PBE function of generalized gradient approximation (GGA); this way, the charge density gradient was introduced to correct the local change [21]. The interaction between valence electrons and ions was described by ultrasoft pseudopotential. Firstly, the structures of SnO_2_ systems were optimized by using the BFGS algorithm. Secondly, the elastic constants of the optimized cell structures were simulated and calculated after reaching the stable state. All the calculation processes were carried out in the reciprocal space. The cut-off energy of plane wave was set as 340 eV, the k-space grid point in the Brillouin zone was set to be 5 × 3 × 6. The convergence criterion was set as follows: the unit electron energy was not higher than 10^−5^ eV/atom, the interaction force between atoms was lower than 0.3 eV/nm, the maximum internal stress was 0.05 GPa, and the maximum displacement convergence accuracy of atoms was 10^−13^ m. The calculated valence electron states were Sn 5s^2^5p^2^, O 2s^2^2p^4^, Cr 3d^5^4s^1^.

### 2.2. Experimental Methods

#### 2.2.1. Materials Preparation Method

**Figure 2:**
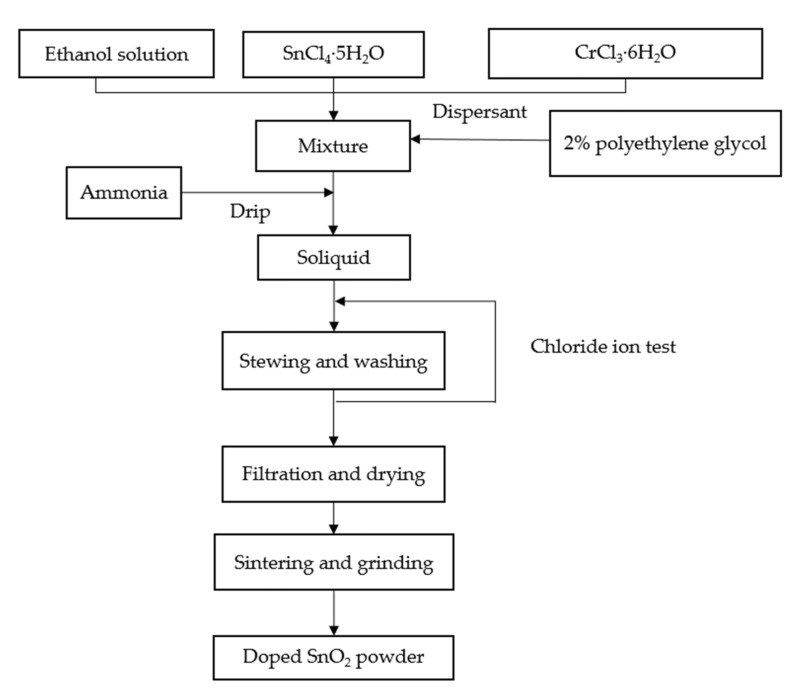
Process of doped SnO_2_ powder preparation by sol-gel method.

For the purpose of correspondence between simulation and experiment, Cr-doped SnO_2_ powders with different ratios were prepared by the sol–gel method, so that the doped ions entered SnO_2_ cells to form solid solutions [22]. The process of doped SnO_2_ powder preparation by the sol–gel method is shown in Figure 2.

Metal halide CrCl_3_·6H_2_O is used for doping raw material and SnCl_4_·5H_2_O is used as a raw material for preparing SnO_2_. First, 50% ethanol deionizing solution was prepared as the solvent, SnCl_4_·5H_2_O and CrCl_3_·6H_2_O were dissolved in the solution, the concentration was 0.2 mol/L. Additionally, 2% of the volume fraction of the mixed solution was added as a drop of polyethylene glycol dispersant to help the powder dissolve. The mixed solution was placed in a DF-101s magnetic mixer (Qiuzuo Scientific Instrument Co., Ltd, Shanghai, China) for 20 min, raised to 70 °C for 30 min before being removed. The following reaction occurred when ammonia water was added dropwise to the solution:

SnCl_4_ hydrolyzed with water:

SnCl_4_ + H_2_O = SnOH^3+^ + H^+^ + 4Cl^−^ (1)

The hydrolyzed Sn^4+^ reacted with OH^−^ in ammonia:

Sn^4+^ + 6OH^−^→ Sn(OH)_6_^2−^ (2)

Sn^4+^ + 4OH^−^ → Sn(OH)_4_↓ (3)

Cr^3+^ + 3OH^−^ → Cr(OH)_3_↓ (4)

Ammonia water was continued to be added dropwise to the mixed solution and floccule formed, but it dissolved quickly.

Sn(OH)_6_^2−^ + 2H^+^ → Sn(OH)_4_↓ + 2H_2_O (5)

Ammonia water was continued to be added dropwise to the mixed solution and the dissolution rate of the formed floccules slowed down and precipitates began to form.

Sn(OH)_4_ → SnO_2_·2H_2_O (6)

2Cr(OH)_3_ → Cr_2_O_3_·3H_2_O (7)

Finally, the doped SnO_2_ gel was prepared. The SnO_2_ gel was dehydrated and washed with Anhydrous ethanol three times after 24 h standing to remove chloridion. Then, the gel was dried (120 °C, 1 h), sintered (500 °C, 1.5 h), and ground to obtain Cr-doped SnO_2_ powders.

**Figure 3:**
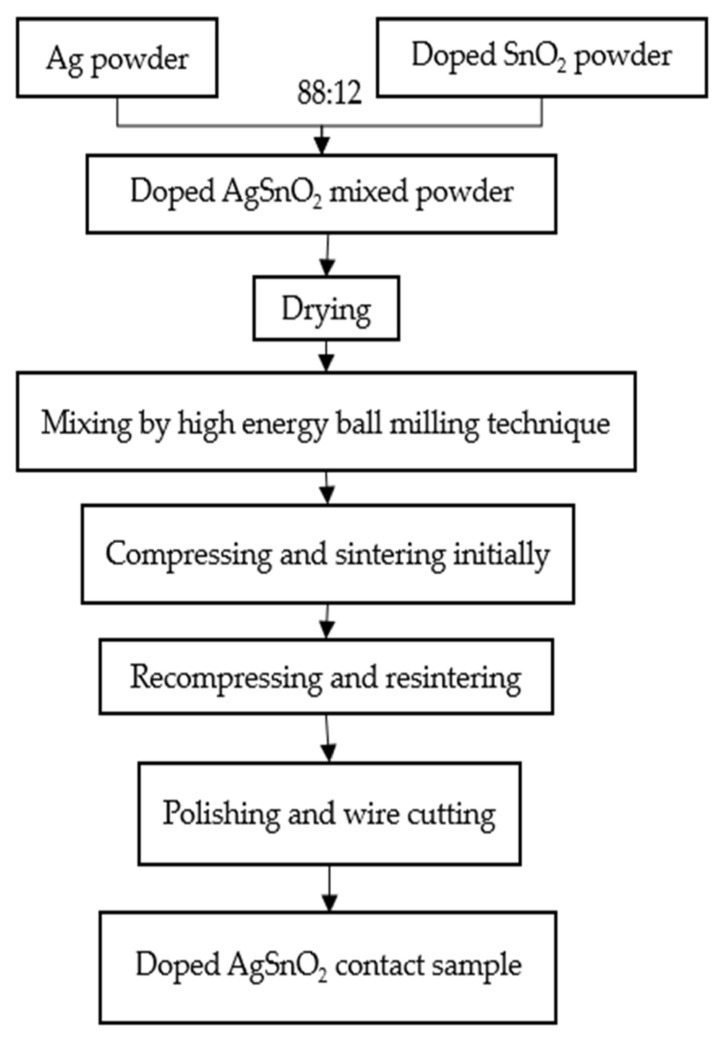
Process of doped AgSnO_2_ contact material preparation by powder metallurgy.

The Cr-doped AgSnO_2_ contact materials were prepared using the high-energy ball milling and powder metallurgy method. This process is shown in Figure 3. The Ag powders and Cr-doped SnO_2_ powders were weighed at a mass ratio of 88:12 and then mixed using the high energy ball milling technique (2 h). The agate balls used for grinding contained twenty balls Φ 10^−2^ m and 10 balls Φ 2 × 10^−2^ m. The ball to powder weight ratio was 15:1. The rounding speed of the ball mill was set as 500 r/min. The mixed powders were compressed into cylindrical samples with a diameter of 20 mm by 769YP-60E powder tablet press machine (Keqi New Technology Co., Ltd, Tianjin, China). The initial pressure was set at 10^7^ Pa (17.6 tf) with 10 min kept. Then, the samples were put into the box-type electric furnace (SIOMM, Shanghai, China) for initial sintering (500 °C, 90 min). In order to enhance compactness, the samples were recompressed (2 × 10^7^ Pa, 10 min) and re-sintered (700 °C, 60 min) to avoid cracks [23]. Finally, the samples were polished and wire cut to smooth the surface. The AgSnO_2_ contact material with a diameter of 3.2 × 10^−3^ m and a thickness of 3.5 × 10^−3^ m was obtained. The instruments and equipment required for the above experiments are shown in Table 2.

#### 2.2.2. X-ray Diffraction Test

In order to verify whether the doped element Cr entered the SnO_2_ lattice and to judge whether the doped SnO_2_ powder prepared in the test was consistent with the simulation model, the Bruker D8 DISCOVERX X-ray diffractometer (Billerica, MA, USA) was used to analyze the phase of the prepared doped SnO_2_ powder. The parameters were set as: the powders were scanned by X-ray with the power of 1.6 kW and the wavelength of 0.15405 nm. The scanning conditions were voltage 40 kV and current 40 mA, and the scanning range 2θ was set as 10°~90°. The scanning speed was 6 °/min.

#### 2.2.3. Measurement of Physical Property

The conductivity of the polished contact material with a diameter of 2 × 10^−2^ m was measured by the Sigmas cope SMP10 metal conductivity tester (Fischer, Baden-Württemberg, Germany) designed according to the eddy current phase principle at a temperature of 20 °C. Each time the conductivity tester was turned on, the probe measurement reference plate was used for calibration first, and then the conductivity of the sample was measured. Each sample was tested three times and their average was taken as a result. The hardness of each contact sample was measured by the HXD-1000TM digital microhardness tester (Precision Scientific Instrument Co., Ltd, Shanghai, China). The instrument can automatically calculate the Vickers hardness of a sample according to the Microindentation method. The sample was placed on the sample table, adjusted until the sample surface could be clearly observed in the eyepiece, and then the machine was operated to make the diamond indenter leave a diamond indentation on the surface of the tested sample. The built-in grating measurement program measured the length of the diagonal and automatically calculated the sample hardness. The sample position was then changed and the above steps were repeated three times. The average value of the three hardness readings were taken as the sample hardness measurement result.

#### 2.2.4. Electrical Contact Experiment Methods

The electrical contact performances of the prepared materials were tested by the JF04D electrical contact material test system (Guiyan Jinfeng Technology Co., Ltd, Kunming, China). The test parameters were set as the DC voltage 20 V, the current 10 A, and the contact pressure 0.86 N. Each pair of contacts was carried out 25,000 on-off tests. In order to keep the contact pressure during the test, the contact materials with a diameter of 3.2 × 10^−3^ m were processed into rivets.

The arc durations and arc energy were measured and recorded by computer. The data recorded were processed by calculating the average value every 100 times.

## 3. Results and Discussion

### 3.1. Simulation Analysis

#### 3.1.1. Crystal Structure and Stability

The lattice constants, volume and enthalpy changes in the Sn_1−x_Cr_x_O_2_ models are shown in Table 3.

The lattice constant and volume of SnO_2_ doping with a Cr increase at different degrees, which is due to the radius and property of the doped atom, are different from that of Sn atom. When Cr enters the lattice by the substitutional doping method, the property and strength of the chemical bond change and atom O moves towards to the doped atom Cr, which causes the equilibrium state of the stress field be destroyed and the arrangement of the atoms changes. As a result, the lattice constant and volume of crystal expand. Furthermore, the radius of Cr^3+^ (0.61 × 10^−10^ m) is slightly smaller than that of Sn^4+^ (0.69 × 10^−10^ m), so the lattice parameters and volumes decrease with the increase in Cr doping concentration. The lattice constant is closest to that of intrinsic SnO_2_ when the doping concentration is x = 0.25.

Enthalpy change is a physical parameter that reflects the difficulty of impurity atoms entering the cell. The generated compound with negative enthalpy change is thermodynamically stable. The greater the absolute value is, the more energy the reaction emits and the more stable SnO_2_ system is. It can be seen from Table 3 that Sn_1−x_Cr_x_O_2_ is thermodynamically stable and the doping scheme is feasible. The absolute value of enthalpy change raises with the reduction in doping concentration. Additionally, the total energy of the system decreases, indicating that the geometrically optimized structure is more stable. The thermal stability enhances with the reduction in doping concentration.

#### 3.1.2. Elastic Constant

Elastic constant is an important mechanical parameter in the study of materials, which can reflect the macroscopic mechanical properties of materials under static loads and test the structural stability of materials. For polycrystalline materials, elastic moduli, such as Young’s modulus, bulk modulus, shear modulus and Poisson’s ratio, can be calculated by elastic constants and then the hardness, stiffness, toughness and other mechanical properties of the material are analyzed therefrom.

For the tetragonal system, there are six independent elastic constants (*C*_11_, *C*_12_, *C*_13_, *C*_33_, *C*_44_, *C*_66_). The criteria to judge the mechanical stability of tetragonal system are shown in the following equation:

(8) $$C_{11}>0,\;C_{33}>0,\;C_{44}>0,\;C_{66}>0,\;C_{11}-C_{12}>0,\;C_{11}+C_{33}-2C_{13}>0,\;2\left(C_{11}+C_{12}\right)+C_{33}+4C_{13}>0$$

The elastic constants of Cr-doped SnO_2_ with different doping ratios are shown in Table 4, and all satisfy the stability criteria. Therefore, SnO_2_ and Cr-doped SnO_2_ with different doping ratios are stable in dynamics. The elastic modulus can be calculated by the elastic constants to analyze the mechanical properties furtherly.

In 1952, Hill proposed that the assumptions of Voigt approximation and Reuss approximation were that polycrystalline material was in equal strain state and equal stress state, respectively [24]. The results obtained by the two approximations were the upper and lower limits of the elastic modulus, respectively. The arithmetic average of them can be used to characterize the elastic modulus of the polycrystalline material. Table 5 shows the Bulk modulus (*B*), shear modulus (*G*), Young’s modulus (*E*), Poisson’s ratio (*ν*), Hardness (*HV*), and universal elastic anisotropy index (*A^U^*) of Sn_1−x_Cr_x_O_2_ calculated by the Voigt–Reuss–Hill method.

The bulk modulus *B* reflects the resistance of material to the external uniform pressure in the elastic system, indicating the incompressibility. The shear modulus *G* represents the ability of the material to resist the shear strain. Young’s modulus *E*, which is an elastic modulus along the longitudinal direction, describes the elastic deformation resistance of solid materials and assesses the stiffness of isotropic elastomers. From Table 4, with the increase in Cr doping concentration, the Bulk modulus (*B*), shear modulus (*G*), and Young’s modulus (*E*) of Sn_1−x_Cr_x_O_2_ increase, indicating the improvement in deformation resistance and stiffness. Poisson’s ratio is an elastic constant, reflecting the transverse deformation of materials. SnO_2_ with the Cr doping ratio of 12.5% has the largest Poisson’s ratio, and the lateral deformation is larger than that of longitudinal deformation after loading. According to the Pugh criterion, the ratio of shear modulus to bulk modulus *G*/*B* can measure the toughness and brittleness of the material, which is closely related to the subsequent processing performance of the material. The higher the brittleness is, the easier fracture occurs during processing. It is generally considered that material with *G*/*B* > 0.57 is brittle, and on the contrary, material with *G*/*B* < 0.57 exhibits toughness. From Table 4, the order of *G*/*B* values is Sn_0.75_Cr_0.25_O_2_ > Sn_0.917_Cr_0.083_O_2_ > Sn_0.833_Cr_0.167_O_2_ > SnO_2_ > Sn_0.875_Cr_0.125_O_2_. When the doping ratio x = 0.125, the *G*/*B* value is the smallest, indicating that it has the best toughness and the strongest ductility.

The hardness reflects the mechanical processing performance of material. The greater the hardness is, the better the wear resistance is, but too high hardness will affect its processing performance, so it is helpful for material to have suitable hardness. With the increase in doping concentration, *HV* of Sn_1−x_Cr_x_O_2_ shows a trend from decline to rise. The universal elastic anisotropy index (*A^U^*) characterizes the difficulty of crack initiation in materials. Except for Sn_0.875_Cr_0.125_O_2_, the *A^U^* values of other SnO_2_ doped systems are lower than that of the intrinsic system, which makes materials more difficult to produce microcracks and plays an important role in improving the properties of materials.

### 3.2. Experimental Results

#### 3.2.1. Phase Analysis by X-ray Diffraction Test

**Figure 4:**
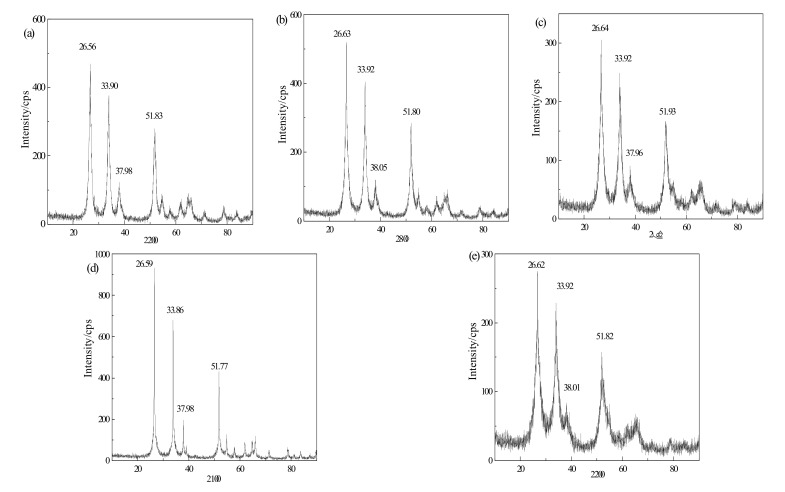
X-ray diffraction pattern. (**a**) x = 0; (**b**) x = 0.083; (**c**) x = 0.125; (**d**) x = 0.167; (**e**) x = 0.25.

Figure 4 shows the X-ray diffraction patterns of intrinsic SnO_2_ and doped SnO_2_ powders, in which the peak positions of the characteristic diffraction peaks are marked. The four representative characteristic diffraction peaks marked in the figures represent the [211], [200], [101] and [110] crystal planes of SnO_2_ from right to left.

Comparing and analyzing Figure 4 a–e, the peak positions of the four characteristic diffraction peaks in the X-ray diffraction pattern of doped SnO_2_ powders prepared by the sol–gel method are basically consistent with the intrinsic SnO_2_ pattern. The offsets are basically 0.1°. The offsets are caused by the change in lattice constant after doping and there is no irrelevant diffraction peak generated by other elements. Therefore, the doped SnO_2_ crystal still belongs to the tetragonal rutile structure, which proves that the doped element Cr enters the SnO_2_ lattice in the form of ion in the preparation process of the sol–gel method, and realizes the integration doping. The doped SnO_2_ powders are consistent with the cell model constructed in the simulation calculation.

#### 3.2.2. Physical Property

The conductivities and hardness of AgSnO_2_ doped with different proportions of elements Cr are shown in Table 6.

The conductivity is promoted after doping according to the experiment data. The contact material has poor compactness without doping, and the conductivities at the existing pores are terrible. The powder particles doped with Cr are smaller and easy to be dispersed in pores. The number and size of pores are reduced, which brings about the compactness and conductivity of contact materials. With the increase in doping concentration, the conductivity increases initially, followed by a descent, reaching the peak at 16.7% doping concentration.

Hardness is closely related to the machining performance of AgSnO_2_ contact material. High hardness contact material has strong mechanical wear resistance but is averse to the processing and forming of the contact meanwhile, which will lead to high brittleness and poor ductility and will easily fracture under the action of an external force. Therefore, it needs to be comprehensively considered according to the actual application environment of the contact material. It can be seen from the data in Table 5 that the hardness of each contact sample is much higher than the national standard, 68 HV, of the hardness of AgSnO_2_ contact material. The reason for the experimental data is that the nano SnO_2_ powders prepared by the sol–gel method in this paper have fine grains, which are easier to flow, diffuse and combine in the preparation process, and are evenly dispersed in the Ag matrix, reducing the air gap and improving the compactness. The experimental data are basically consistent with the simulation results. When the doping ratio is 25%, the hardness of Cr-doped AgSnO_2_ contact material is the highest. On the contrary, the hardness of Sn_0.875_Cr_0.125_O_2_ is the lowest.

#### 3.2.3. Electrical Contact Property

The electrical contact performance of contact materials can be evaluated from two indices: arc duration and arc energy.

The high temperature arc is generated during the on-off process of the electric contact, which produces a lot of Joule heat during arc combustion, resulting in the temperature rising to the melting point of the contact Ag matrix, causing metal splash and loss, as well as ablation of the electric contact material. Arcing duration, which is an important parameter for estimating the electrical properties of contact materials, refers to the time interval from arc starting to arc extinguishing. It has a major impact on arc energy and contact ablation. The longer the arc duration is, the more serious the contact ablation is. The main factors affecting contact arcing time are contact surface state and contact material composition.

**Figure 5:**
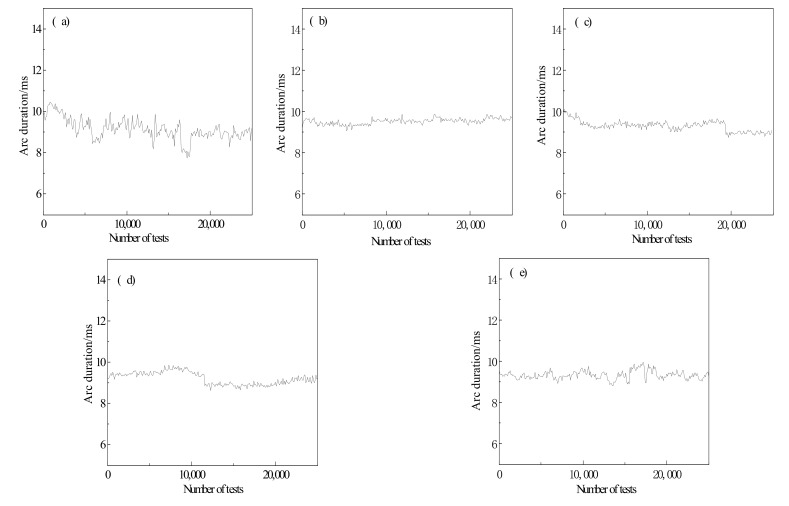
Arc duration of Sn_1−x_Cr_x_O_2_ contact material. (**a**) x = 0; (**b**) x = 0.083; (**c**) x = 0.125; (**d**) x = 0.167; (**e**) x = 0.25.

The variation curves of arc duration of Cr-doped AgSnO_2_ contact materials are shown in Figure 5. In the early stage of the experiment, the surface of the AgSnO_2_ contact is rough, so the arc concentrates on the protruding small area between the two contacts; as a result, the arc burning time is long. As the test is carried out, first, the contact surface deteriorates and the arc duration prolongs under arc erosion; second, the original uneven contact area is burned and smoothed by the arc, leading to shorter arc duration. In the process of arc erosion, the two factors work together. In different periods, a certain factor plays a significant role, resulting in the fluctuation of the arc duration curve. The arc duration curve of undoped AgSnO_2_ contact material fluctuates violently, indicating that the arc erosion resistance of the contact is unstable. From Table 7, it can be seen that the average arc duration of AgSnO_2_ contact materials doped with different proportions of Cr appears a trend from decline to rise. When the doping ratio is 16.7%, the average arcing time is the smallest.

Arc energy, which generates by high-temperature arc combustion, is affected by arc duration and arc-burning resistance. The higher the arc-burning energy is, the easier it is to rise contact temperature to the melting point of the contact material, which brings about the ablation of electrical contact and splash and loss of material.

**Figure 6:**
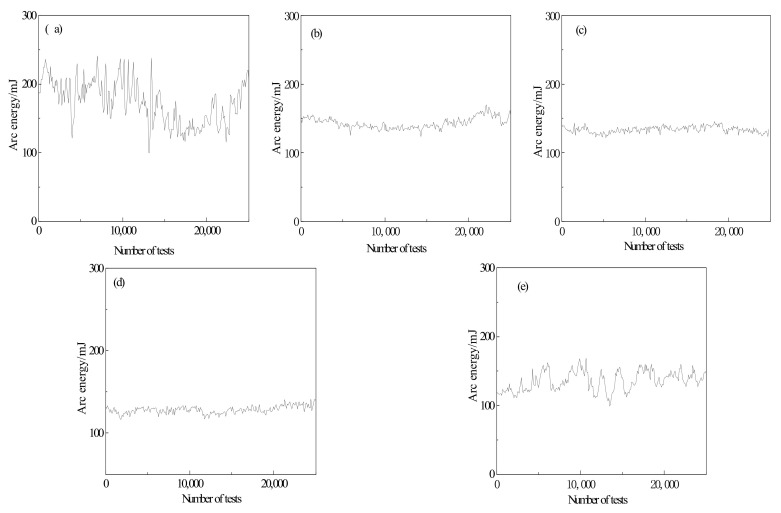
Arc energy of Sn_1−x_Cr_x_O_2_ contact material. (**a**) x = 0; (**b**) x = 0.083; (**c**) x = 0.125; (**d**) x = 0.167; (**e**) x = 0.25.

The variation curves of arc energy of Cr-doped AgSnO_2_ contact materials are shown in Figure 6. It can be seen from the data in Table 6 that the trend of arc energy of each contact material is consistent with arc duration. When the doping concentration is 16.7%, the arc erosion resistance of the contact material is the best. For the undoped contact, the arc energy fluctuates violently, and the arc energy is much higher than that after doping. Additionally, the heat flux per unit area of contact arc area is high, resulting in serious arc erosion and more possibility of fusion welding of the contact. The doped AgSnO_2_ contact with good thermal stability can maintain the doped SnO_2_ suspended in molten metal liquid pool formed by arc action, effectively inhibit aggregation of doped SnO_2_ on the contact surface, improve the wettability of silver on the contact, reduce splash loss of Ag, inhibit arc ablation of contact, and improve the arc erosion resistance of AgSnO_2_ contact material.

The metal doping element Cr can stabilize arc root, hindering the movement of arc root spots to the edge region, contributing to the slight fluctuation of arc energy and stable performance of AgSnO_2_ contact material. The arc energy curve has an increasing trend in the late stage of the test. This is because, along with the test, under the repeated thermal action of arcs, a large amount of Joule heat is generated, resulting in temperature rise in contact surface, material splash and loss, worse deterioration and erosion of contact surface, which intensifies arc combustion and heightens arc energy.

However, the study had some limitations in the combination of simulation and experiment; the simulation part could not correspond to the electrical contact experiment. In addition, the tensile strength test was not conducted due to the lack of test equipment. Furthermore, the electrical contact experiment was only a simulated experiment, without reliability tests and other experiments in the actual industrial environment.

## 4. Conclusions

Based on the first principle, the elastic constants of Sn_1−x_Cr_x_O_2_ were calculated in the paper to analyze mechanical properties. The Cr-doped AgSnO_2_ contacts with different doping proportions were prepared by the sol–gel and powder metallurgy method. The hardness, conductivities and electrical contact properties of contacts were tested.

The simulation results show that among doped SnO_2_ supercell models, the Bulk modulus (*B*), shear modulus (*G*), and Young’s modulus (*E*) of Sn_1−x_Cr_x_O_2_ increase with the rise of Cr doping concentration, indicating the improvement of deformation resistance and stiffness. The value of *G*/*B* and *HV* are the smallest, with a doping ratio of 12.5%, characterizing that the material has the best toughness and the strongest ductility, leading to better processing capability and the slightest inclination to fracture during processing.

The experimental results show that, the doped SnO_2_ powders belong to the tetragonal rutile structure. The doped element enters the SnO_2_ lattice in the form of ions, realizing the integration doping, which is consistent with the crystal model constructed in the simulation calculation. The hardness test results correspond to the simulation results. When the doping ratio is 12.5%, Cr-AgSnO_2_ has the lowest hardness. Compared with undoped AgSnO_2_, the conductivity of Cr-doped contact material increases, the arc duration and arc energy decrease, the arc root is stabilized to inhibit the arc ablation of contact, and the electrical contact performance is stable and excellent. When doping concentration is 16.7%, the Cr-doped AgSnO_2_ contact material has the best conductivity and arc erosion resistance.

## Figures and Tables

**Table 1:** Doping ratio and supercell correspondence.

Supercell	Doping Ratio (%)
1 × 1 × 1	0
1 × 2 × 3	8.33
1 × 2 × 2	12.5
1 × 1 × 3	16.7
1 × 1 × 2	25

**Table 2:** Experimental equipment.

Equipment	Type	Manufacturer	Purpose
Digital balance	DT200A	Precision Scientific Instrument Co., Ltd, Shanghai, China	Weighing chemical raw materials
Constant temperature magnetic mixer	DF-101S	Qiuzuo Scientific Instrument Co., Ltd, Shanghai, China	Stirring the solution to dissolve the solute
Vacuum pump suction filter	FY-1C-N	Jingmai Instrument Equipment Co., Ltd, Shaoxing, China	Filtering and dewatering
Vacuum drying oven	DZ-1BCLV	Taisite Instrument Co., Ltd, Tianjin, China	Drying
Precision box type test electric furnace	SXL-1200	SIOMM, Shanghai, China	Sintering
Omnidirectional planetary mill	QXQM-2	Tianchuang Powder Technology Co., Ltd, Changsha, China	Mixing powders
Powder tablet press	769YP-60E	Keqi New Technology Co., Ltd, Tianjin, China	Compressing
polish-grinding machine	YMPZ-2A-250	Metallurgical Machinery Equipment Co., Ltd, Shanghai, China	grinding and polishing

**Table 3:** Lattice constant, volume and enthalpy change.

Doping Ratio	*a* (10^−10^ m)	*b* (10^−10^ m)	*c* (10^−10^ m)	Volume ((10^−10^ m)^3^)	Δ*H* (eV)
0	4.74	4.74	3.18	71.51	−0.012
0.083	4.87	4.88	3.24	78.19	−4.111
0.125	4.87	4.85	3.26	77.23	−2.889
0.167	4.82	4.82	3.25	76.26	−2.281
0.25	4.79	4.79	3.23	74.24	−1.704

**Table 4:** Elastic constants.

Doping Ratio	*C*_11_	*C*_12_	*C*_13_	*C*_33_	*C*_44_	*C*_66_
0	184.09	111.62	100.48	339.49	81.59	165.08
0.083	176.19	95.21	109.91	191.25	78.37	78.31
0.125	183.54	108.10	111.26	179.49	81.21	81.04
0.167	319.52	−13.43	105.57	354.73	92.41	42.58
0.25	318.35	−15.79	103.27	358.59	101.91	44.03

**Table 5:** Bulk modulus (*B*), shear modulus (*G*), Young’s modulus (*E*), Poisson’s ratio (*ν*), Hardness (*HV*), and universal elastic anisotropy index (*A^U^*) of various SnO_2_ systems.

Doping Ratio	*B* (10^9^ Pa)	*G* (10^9^ Pa)	*E* (10^9^ Pa)	*G/B*	*ν*	*HV*	*A^U^*
0	144.059	82.102	206.984	0.569	0.261	11.003	1.427
0.083	139.952	80.489	202.623	0.575	0.259	10.962	1.353
0.125	146.609	81.638	206.572	0.557	0.265	10.673	1.599
0.167	151.994	86.800	218.758	0.571	0.260	11.471	1.176
0.25	152.102	89.385	224.231	0.588	0.254	12.099	1.164

**Table 6:** Physical property of contact materials.

Doping Ratio	Conductivity (10^6^ S × m^−1^)	Hardness (HV)
0	24.39	117.14
0.083	27.13	110.59
0.125	26.99	106.33
0.167	29.41	119.07
0.25	25.13	121.86

**Table 7:** Arc duration and arc energy.

Doping Ratio	Average Arc Duration (ms)	Average Arc Energy (mJ)
0	9.600	173.783
0.083	9.528	142.949
0.125	9.304	133.656
0.167	9.221	128.520
0.25	9.339	135.718

## Data Availability

Not applicable.
